# Preclinical evaluation of Sunitinib as a single agent in the prophylactic setting in a mouse model of bone metastases

**DOI:** 10.1186/1471-2407-13-32

**Published:** 2013-01-24

**Authors:** Christian Schem, Dirk Bauerschlag, Sascha Bender, Ann-Christin Lorenzen, Daniel Loermann, Sigrid Hamann, Frank Rösel, Holger Kalthoff, Claus C Glüer, Walter Jonat, Sanjay Tiwari

**Affiliations:** 1 Molecular Imaging North Competence Center, Department of Diagnostic Radiology, University Hospital Schleswig-Holstein, Campus Kiel, Germany; 2 Division of Molecular Oncology, Institute for Experimental Cancer Research, University Hospital Schleswig-Holstein , Campus Kiel, Germany; 3 Department of Gynecology, University Hospital Schleswig-Holstein, Campus Kiel, Germany; 4 Department of Gynecology, University Hospital Aachen, Aachen, Germany

**Keywords:** Sunitinib, Bone metastases, Breast cancer, Imaging

## Abstract

**Background:**

A substantial number of breast cancer patients are identified as being at high risk of developing metastatic disease. With increasing number of targeted therapeutics entering clinical trials, chronic administration of these agents may be a feasible approach for the prevention of metastases within this subgroup of patients. In this preclinical study we examined whether Sunitinib, a multi-tyrosine kinase inhibitor which has anti-angiogenic and anti-resorptive activity, is effective in the prevention of bone metastases.

**Method:**

Sunitinib was administered daily with the first dose commencing prior to tumor cell inoculation. Intracardiac injection was performed with MDA-MB23 bone-seeking cells, which were stably transfected with DsRed2. In vivo plain radiography and fluorescent imaging (Berthold NightOwl) was used in the analysis of bone metastases. Histomorphometry was used for the quantification of TRAP^+^ cells from bone sections and immunohistochemistry was performed using an antibody reactive to CD34 for quantification of microvessel density.

**Results:**

Preventive dosing administration of Sunitinib does not inhibit colonization of tumor cells to bone or reduce the size of osteolytic lesions. There was a decrease in the number of TRAP^+^ cells with Sunitinib treatment but this did not reach significance. Sunitinib inhibited tumor growth as determined by imaging of fluorescent tumor area. Immunohistochemical analyses of microvessel density revealed a concomitant decrease in the number of tumor blood vessels.

**Conclusions:**

The findings suggest that Sunitinib can be used as a therapeutic agent for the treatment of bone metastases but as a single agent it is not effective in terms of prevention. Therefore a combination approach with other cytostatic drugs should be pursued.

## Background

Up to 40% of patients with early stage breast cancer have disseminated tumors cells in the bone marrow [1]. Furthermore, bone is the most common site for breast cancer metastasis with 50% of metastatic breast cancer patients presenting with bone metastasis. Increased bone resorption is becoming increasingly recognized as a risk factor for development of metastatic tumor in the bone. A number of preclinical studies have demonstrated that heightened bone resorption creates an environment that promotes growth of breast and prostate cancer cells [2-7]. Therefore, agents that are anti-resorptive may prevent the development of bone lesions and reduce the risk of relapse in bone. Indeed, clinical trials have shown that adjuvant anti-osteoclast therapy with the bisphosphonates Zoledronic Acid results in decreased numbers of disseminated tumor cells in the bone marrow [8,9]. Furthermore, adjuvant Zoledronic Acid therapy in post-menopausal women with early stage breast cancer results in an increase in relapse free survival [10,11].

Preclinical studies indicate that angiogenic inhibitors are effective in reducing tumor burden in bone [12-15]. The mode of action is through dual targeting of tumor blood vessels and osteoclast activity [12,14,16-18]. Inhibition of VEGF signaling can markedly affect bone remodeling since VEGF directly affects the proliferation and maturation of osteoclasts, osteoblasts, and their precursors [19-24]. However, their effectiveness in reducing metastatic risk from disseminated tumor cells is unknown. Amongst the patients who may benefit from prophylactic treatment are those who have higher numbers of involved lymph nodes, larger tumor size, a triple negative phenotype and/or those with a low estrogen, high bone resorptive environment.

For a therapeutic approach in the prophylactic setting to be rational, the angiogenic inhibitor should be cytostatic since non-tumorigenic endothelial cells and osteoclasts are targeted. In addition, the toxicity profile should be supportive of long-term chronic administration of the drug. Sunitinib malate is a small tyrosine kinase inhibitor with antiangiogenic activity and a safety profile which has been reported to be acceptable for chronic outpatient therapy [25]. The predetermined efficacious dose of 40 mg/kg daily in mice maintains plasma Sunitinib concentration above 50 ng/mL, selectively inhibiting VEGR2 and PDGF receptor phosphorylation [26]. In clinical trials, dosing that gives rise to Sunitinib plasma concentration of equal or greater than 50 ng/mL was determined to be 50 mg/day [27,28]. Recent phase III clinical trials have revealed that administration of Sunitinib to patients with advanced breast cancer did not increase progression free survival or overall survival when used either as a single agent or in combination with chemotherapeutic agents [29,30]. In this study we tested the hypothesis that administration of a clinically relevant dose of Sunitinib malate in the prophylactic setting will decrease the number and extent of osteolytic bone lesions. We show efficacy with Sunitinib monotherapy in inhibiting tumor growth of bone metastases but not the number and size of osteolytic lesions.

## Methods

### Tumor cell inoculation

Bone-seeking MDA-MB231 cell line (MDA-231BO) was obtained from Dr Toshiyuki Yoneda (University of Texas Health Science Center). The cell line was stably transfected with the fluorescent reporter gene DsRed2 (Clontech) and selected using 800 μg/ml of neomycin. Following establishment of the stably transfected cell line (MDA-231BO-DsRed2), three further in vivo passages to the bone were performed. One hundred thousand MDA-231BO-DsRed2 cells were injected by ultrasound guidance into the left cardiac ventricle of 6 week old female Fox nu/nu mice. Animal experiments and care were in accordance with the guidelines of institutional authorities and approved by local authorities (number, V 312–72241.121-10 (53-5/06).

### Treatment groups

All treatment began two days prior to tumor cell inoculation. One group of mice (n = 7) were treated with 40 mg/kg Sunitinib (orally/daily), a second group served as a control group and was administered with carboxymethylcellulose vehicle formulation (orally/daily) (n = 7).

### Fluorescent imaging

Anesthetized mice were imaged for DsRed2 fluorescence using a Peltier cooled charged-coupled device camera (NightOWL LB 983; Berthold Technologies, Bad Wildbad, Germany) to assess tumor growth at weeks 3, 4 and 5 post-inoculation. The excitation source is a ring light used for epi-illumination, mounted 12 cm above the mice. Filters of 550 nm (±10 nm) and 605 nm (±55 nm) were used to assess excitation and emission signals respectively. The exposure time was set to 5 s. Using the WinLight 32 software (Berthold), fluorescent signals (expressed in counts/s) from the images were calculated by selecting a rectangular region of interest around the tumor and integrating the signal of each pixel over the chosen area. To account for variations in autofluorescence over time and between mice, the rectangular region of interest was placed over an adjacent non-bone area to determine the background signal. This signal was then subtracted from the tumor signal. The tumor area was calculated using the Winlight32 software and expressed as mm^2^. The threshold of fluorescence emission was set to the level at which non-specific fluorescent signal was no longer detected in adjacent skin. This operated only on the periphery of the tumor, after which an automated peak search function was utilized to delineate the area of the fluorescent tumor.

### Radiographs

Anesthetized mice were radiographed using a LoRad Selenia digital mammography unit (Hologic GmbH, Frankfurt/Main, Germany). Radiographs were taken at weeks 3, 4 and 5 following intracardiac injection and each radiograph was blindly evaluated by CS and A-CL. The area of osteolytic lesions was measured using a computerized image analysis system (IMPAX; AGFA, Cologne, Germany) and results were expressed in square millimeters.

### Histomorphometry

Hind limbs from each animal were dissected and fixed in neutral buffered formalin overnight at +4°C. The bone samples were washed for two hours in cold PBS and decalcified in acetic acid for 6 hours at +4°C. Following decalcification the biopsy sample was embedded in paraffin wax cut into 7 μM sections using a microtome. Histomorphological analyses of the proximal tibia or the distal femur were performed by cutting a series of 70 μm sagittal sections every 200 μm, corresponding to the upper, mid and lower regions and 3 sections were analysed for each animal. The sections were then de-paraffinized and stained with H&E, TRAP (tartrate-resistant acid phosphatase), and CD34. TRAP staining was performed according to van de Wijngaert and Burger [31] following de-paraffinization and rehydration of the sections in serial alcohol dilutions. Briefly, the sections were washed in distilled water and incubated for 20 min in a solution containing 0.2 M Sodium Acetate (Merck, Catalog Nr. 6268) and 50 mM Tartaric Acid, pH 5.0 (Sigma-Aldrich, Catalog Nr. T10-9). The sections were then incubated in the TRAP running buffer containing 0.1mg/ml napthol AS-MX phosphate (Sigma N4875) and 1.1 mg/ml Fast Red TR (Sigma, F8764-16) for 1–3 h at 37°C until colour reaction was complete. The sections were washed in distilled water, stained with haematoxylin and mounted. TRAP enumeration was performed by selecting the tumor region in the distal femur or proximal tibia and counting the number of TRAP-positive cells in contact with endosteal surface. Five random regions were selected for counting and data are expressed as the number of TRAP^+^ / bone surface / mm^2^. CD34 staining was performed using the Anti-Rat HRP-DAB staining kit (R&D Systems, Catalog Nr. CTS017) with a rat monoclonal anti-CD34 antibody (Gene Tex, Catalog Nr. GTX 28158) at 1:500 dilution (50 μg/ml). Microvessel density (MVD) was evaluated by calculating the selecting three highest areas of vascularity across the tumor region and the number of angiogenic vessels were counted in each field of view. The number of vessels counted was divided by the field of view to yield the MVD, expressed as MVD/mm^2^. Histomorphometric measurement of tumor area was performed using the longitudinal section of the distal femur. Sections through the tumor comprising of the largest tumor area were stained with Goldners trichrome to identify tumor area and structural organization of the bone. Tumor area was measured from the epiphyseal line of the growth plate and extending into the diaphysis and bilaterally between the endocortical surfaces. A line was drawn around the tumor margin using Image J software and a scale bar was used to measure lengths and calculate the cross-sectional area. At regions of extensive cortical destruction, the tumor area included growth to the boundary of the periosteum.

### Statistical analyses

Two-tailed unpaired Student’s t tests were used to assess differences between treated and control groups. P values less than 0.05 were considered statistically significant. All statistical analyses were performed using GraphPad Prism 4.03.

## Result

### Preventive dosing administration of Sunitinib does not inhibit colonization of tumor cells to bone

Intra cardiac injection of tumor cells recapitulates the later stages of metastases whereby tumor cells that metastasize to the skeleton adhere to the endosteal surface and colonize bone. To determine if preventive Sunitinib treatment reduced establishment of colonized sites, mice were administered with Sunitinib at the previously established efficacious dose of 40 mg/kg/day. Dosing commenced two days before the mice were inoculated with tumor cells. Since tumor cells stably expressed the red fluorescent protein DsRed2, the number of fluorescent spots corresponding to metastatic boney sites of the mandible, hind legs, spine and ribs were counted. Imaging at three weeks revealed no detectable bone metastases but by four weeks a number of metastases were visible. No significant difference in the number of bone metastases was observed with Sunitinib treatment compared to the control group (Table 1).

**Table 1:** Total number of bone metastases in control and Sunitinib groups

**Group**	**Mice Nr.**	**Total Nr.of metastasis at 4 weeks**	**Total Nr.of metastasis at 5 weeks**
Control	7	14	21
Sunitinib (40 mg/kg/day)	7	12	21

Fluorescent bone metastatic lesions were counted at week 4 and week 5 following tumor cell inoculation. No significant differences in the mean number of metastases was observed between groups.

### Sunitinib does not reduce the size of osteolytic lesions

**Figure 1:**
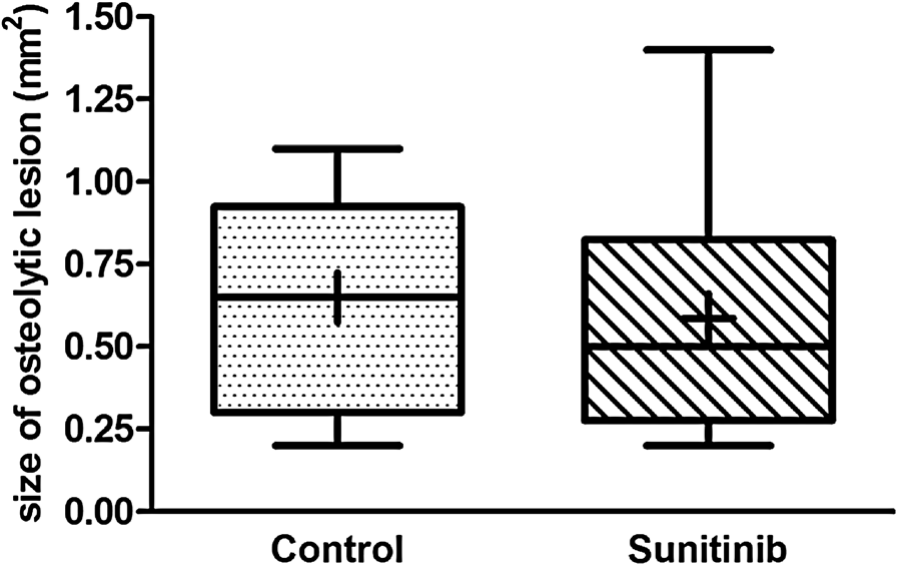
**Size of osteolytic lesions (mm^**2**^)in nude mice with bone metastases.** Osteolytic lesions were analysed by plain radiography 5 weeks following tumor cell inoculation; Control (n = 7), Sunitinib (n = 7). Box plot shows 25–75 percentiles, whiskers 05–95 percentiles,+ indicates mean and line indicates median. Significance was determined by 2-tailed Student t-test (*p < 0.05, ^o^p < 0.1). No difference is observed in the size of osteolytic lesions with Sunitinib therapy (mean ± SD: Ctrl 0.65 ± 0.33; Sunitinib 0.59 ±0.35).

To determine if preventive treatment with Sunitinib reduced the size of osteolytic lesions, plain radiography imaging of leg and rib metastases was performed at week 5 following tumor cell inoculation and the size of the osteolytic lesions was measured (Figure 1). Sunitinib treatment did not significantly decrease the size of osteolytic lesions compared to the control group.

**Figure 2:**
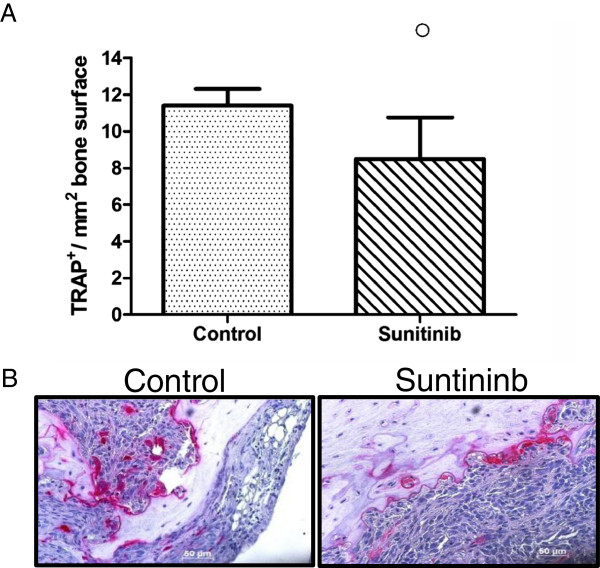
**(A) Number of TRAP**-**positive osteoclasts at the bone**-**tumor interface.** Six weeks after tumor cell inoculation bones were fixed in formalin, decalcified in acetic acid and embedded in paraffin. Sections were stained for TRAP and counterstained for Haematoxylin. Five fields of tumor for each specimen were randomly selected and counted to determine the number of TRAP-positive multinucleated cells. Column bar depicts mean ± SD of 5 animals per group. ^o^p < 0.1 (**B**) Representative sections of bone metastases stained for TRAP (Red) for detection of osteoclast and counterstained with Haematoxylin (blue) for detection of tumor cells. Magnification 20x.

To determine the effect of treatment on osteoclast number, bone sections of the hind legs were stained for TRAP to determine presence of osteoclasts at the tumor-bone interface. Histological examination showed lower TRAP-positive osteoclasts in the metastatic hind legs of mice treated with Sunitinib but this difference was not significant (Figure 2A). Histological appearances of representative sections stained for TRAP are shown in Figure 2B.

### Sunitinib as a monotherapy inhibits tumor growth

**Figure 3:**
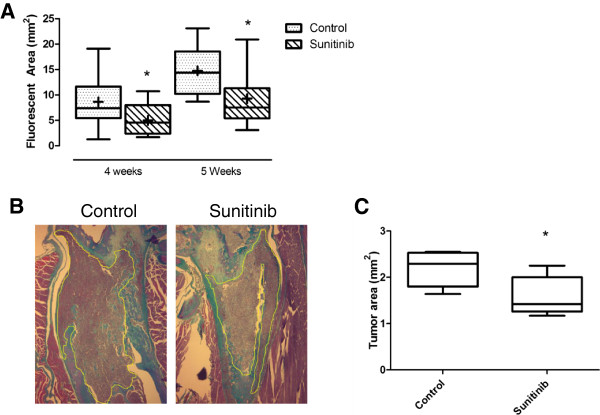
**(A) In vivo imaging of fluorescent tumor area to monitor tumor growth.** Nude mice were injected with 1 x 10^5^ MDA-231BO-DsRed2 cells into the left ventricle and fluorescent imaging was performed at regular intervals. Analyses of fluorescent tumor area was performed for Control (n = 7), Sunitinib (n = 7). Box plot shows 25–75 percentiles, whiskers 05–95 percentiles, + indicates mean and line indicates median. Significance was determined by 2-tailed Student t-test (*p < 0.05). Growth inhibition with Sunitinib treatment is significantly reduced at 4 and 5 weeks following tumor cell inoculation (mean ± SD: Week 4. Ctrl 8.62 ± 5.13; Sunitinib 4.99 ±3.00. Week 5. Ctrl 14.75 ± 4.97; Sunitinib 9.30 ± 5.42). (**B**) Histomorphometric determination of cross-sectional tumor size. Longitudinal sections of distal femur were stained with Goldners trichrome and the tumor area measured from the epiphyseal line associated with the growth plate and extending into the diaphysis and bilaterally between the endocortical envelope. The tumor margin measured is marked by a green line. At regions of extensive cortical destruction, the line was drawn to the boundary of the periosteum. Representative sections of Goldner’s trichrome stained femur from control and Sunitinib treated mice are shown. Magnification 5x (**C**) Cross-sectional tumor size in bone as measured by histomorphometry is significantly decreased with Sunitinib treatment (Control mean ± SD 2.19 ± 0.39, n = 5; Sunitinib mean ± SD 1.59 ± 0.43; n = 5, p < 0.05).

The effect of Sunitinib on tumor growth as a single agent was evaluated. Fluorescent tumor area was determined as this parameter has been reported to have a better correlation with micro-CT-based osteolytic lesion grade than fluorescent intensity [32]. The fluorescent area of metastatic tumors was measured when tumors were first detected at 4 weeks following tumor cell inoculation and again one week later. Mice treated with Sunitinib alone had significantly lower tumor fluorescent area at 4 weeks than mice in the control group (Figure 3, Mean ± SD: 8.62 ± 5.13 vs 4.99 ± 3.00, p < 0.05). Furthermore, imaging at 5 weeks also revealed significantly smaller tumors (Mean ± SD: 14.75 ± 4.97 vs 9.30 ± 5.42, p < 0.05). Therefore Sunitinib preventive treatment results in inhibition of growth of smaller tumors in bone and is also effective in inhibiting tumor growth of established tumors in the bone microenvironment. Histological measurement of cross-sectional tumor area from the epiphyseal line of the distal femur and extending into the diaphysis confirmed a decrease in size of tumor in the bone with Sunitinib treatment (Figure 3B & C).

**Figure 4:**
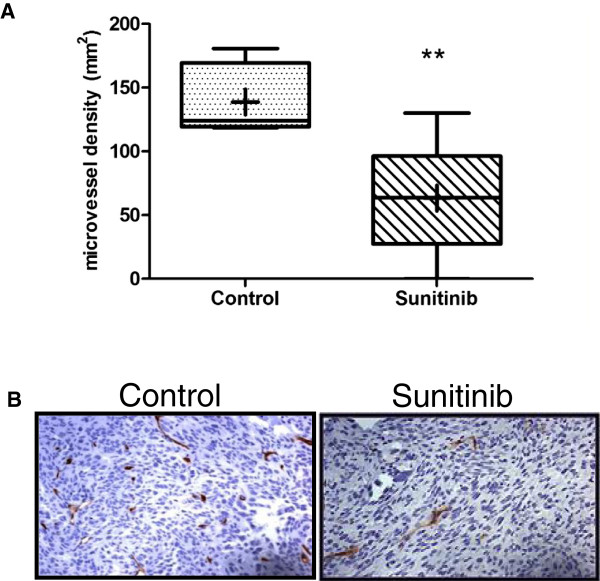
**(A) Immunohistochemical staining of blood vessel density was performed on bone sections using the pan**-**endothelial marker CD34 and quantified.** Three regions with highest blood vessel density were selected and the number of angiogenic vessels counted. Box plot shows 25–75 percentiles, whiskers 05–95 percentiles, line indicates median of 5 animals per group. Significance was determined by 2-tailed Student t-test (**p < 0.01). Blood vessel count is significantly reduced with Sunitinib treatment (mean ± SD: Ctrl 138.8 ± 27.3; Sunitinib 63.4 ± 45.7). (**B**) Representative staining of CD34+ blood vessels (brown) and counterstained with Haematoxylin (blue). Magnification 200x.

Furthermore, histological evaluation of tumor angiogenesis revealed that Sunitinib significantly inhibited tumor neovascularization. The mean vessel density (MVD) of hind legs with bone metastases was 63 ± 17 with Sunitinib treatment compared to 139 ± 11 for control mice (p < 0.01, Figure 4A). The data suggest that Sunitinib is effective in inhibiting growth of bone metastases through inhibition of new blood vessel formation. Histological appearances of representative sections stained for CD34 are shown in Figure 4B.

## Discussion

In this study we evaluated whether preventive dose administration of the multi-tyrosine kinase Sunitinib is effective in inhibiting bone metastases arising from disseminated tumor cells. We show that Sunitinib used in the prophylactic setting does not decrease the number of metastatic colonized sites or inhibit subsequent progression of osteolytic lesions. There is however a reduction in tumor growth which is associated with a significant decrease in tumor blood vessels. The findings suggest that Sunitinib alone is not able to prevent colonization and expansion of disseminated tumor cells to bone but may be a useful adjunctive therapy in reducing metastatic tumor burden.

It has been reported that prior administration of Sunitinib at elevated doses (120 mg/kg/day) induce a "conditioning effect" which promote the formation of metastasis by circulating tumor cells [33]. In this study pre-treatment of mice with a dose of 40 mg/kg/day did not lead to enhanced metastasis. This observation is consistent with the findings of others that have used lower therapeutically efficacious doses [34,35] and support a rationale for application of lower doses of Sunitinib to avoid augmented invasive or metastatic potential.

The concept of the vicious cycle of bone metastases emphasizes the cross-talk between tumor cells and the bone microenvironment in the process of tumor growth and bone destruction [36,37]. Following colonization to the bone, secretion of factors by tumor cells stimulate osteoclastogenesis via upregulation of receptor activator of nuclear factor kappa B ligand (RANKL) on osteoblast and stromal cells. The increased bone resorptive activity releases growth factors from the bone matrix which in turn stimulate tumor cell growth giving rise to further bone destruction. The observation that Sunitinib did not show any therapeutic efficacy in inhibiting osteolytic lesions despite a reduction in tumor size suggest that osteoclast proliferation and activity was sufficiently stimulated through secretions of growth factors by the tumor. Increased osteolyses associated with tumor shrinkage and decreased vascularization was described previously for the treatment of bone metastases using the histone deacetylase inhibitor Vorinostat [38]. The effect was attributed to off-target effects of the drug on a sub-population of resistant cells giving rise to increased secretion of factors promoting osteolysis. Sunitinib has recognized off-target activity with one report indicating binding to at least 5 off-target kinases with high affinity [39]. A possible off-target effect following Sunitinib therapy is the tumor-independent increase in systemic levels of factors such as granulocyte colony-stimulating factor and osteopontin [40], factors which are known to promote bone resorption [41-46]. In this regard, the application of second-generation tyrosine kinase inhibitors (TKI's), such as pazopanib and tivozanib, with improved potency and selectivity may provide more effective treatment options [47,48]. Another possibility which may explain our observation is the adaption of an evasive response by MDA-MB231 cells. The cell line is of metastatic origin and an evasive response to disturbed tumor vasculature may be manifested by increased secretion of certain factors which induce osteolysis. Adaptive evasive responses leading to increased invasiveness and distant metastases have been observed in mouse models of pancreatic neuroendocrine carcinoma and glioblastomas following antiangiogenic targeting of VEGF signaling [49,50]. The mechanism by which this occurs is not understood but the hypoxia/HIF-1α pathway is implicated.

With an observed decrease in tumor growth in bone there is a compelling biological rationale for the use of targeted combination therapy with an anti-resorptive agent. A retrospective study of patients with bone metastases from renal cell carcinoma revealed an increase in progression free survival and response rate in patients treated concomitantly with bisphosphonate and Sunitinib [51]. Amongst targeted agents which inhibit osteoclast activity and are currently subject to clinical evaluation in breast cancer bone metastases are inhibitors to mTOR, RANKL, Src and Cathepsin K. A recent clinical trial administrating the mTOR inhibitor Everolmus, a rapamycin derivative which inhibits mTORC1, showed beneficial effects on bone turnover and bone progression in postmenopausal women with oestrogen-receptor-positive breast cancer [52]. Denosumab, a human neutralizing antibody to RANKL, has been shown to have a stronger inhibitory effect on bone resorption than bisphosphonates [53-55]. Since long term Denosumab therapy is generally well tolerated, introduction of Denosumab as a second agent for the inhibition of osteoclast activity, may further deprive tumor cells of growth factors sequestered in the bone matrix. In view of the fact that bone remodeling is a complex process requiring orchestrated differentiation of different cell types as well as angiogenesis, establishment of treatment order of agents and dosage may influence therapeutic effects of combination treatment [56].

Certain limitations of this study are outlined. One is that only a single cell line was employed which is unlikely to reflect the phenotypic properties of all disseminating cells especially those disseminating from the primary tumor at an early stage of the disease. Secondly, the intracardiac injection model does not recapitulate the requirement for cells to escape the primary site and so not all the steps of the metastatic process can be studied. Thirdly, the use of mouse models in itself has the limitation that ethical time points arrive much earlier than for example in a rat model and it is uncertain whether a longer period of Sunitinib treatment may eventually had an effect on osteolytic size.

With an increasing number of targeted therapies entering clinical trials, there is an urgent need to apply these drugs for the prevention of fully developed metastatic lesions arising from disseminated tumor cells. With advances in molecular biomarkers that refine and improve risk stratification in patients, those with a high risk of developing bone metastases may benefit from adjuvant or prophylactic treatment with targeted therapeutics, as long as the cytotoxicity is supportive of long term chronic administration. The targeted therapies that may come into consideration are those that target genes involved in disseminated tumor cells infiltration, survival and colonization of the bone. In the event that metastases ensue, the drug can then be combined with a cytotoxic agent to invoke additive antitumor activity.

The data presented here establish that Sunitinib inhibits tumor bone growth when applied in the preventive setting. Further preclinical studies are warranted to test the potential combination with an anti-resorptive agent in the prophylactic setting, with the aim to improve their overall antitumor efficacy. With the development of more specific and well tolerated second generation TKIs which inhibit VEGF signaling, the prospect of applying these agents in combination with anti-resorptive agents holds significant promise for the prevention of bone metastatic disease.

## Conclusion

We present the first application of Sunitinib in a preclinical mouse model for the prevention of bone metastases and show efficacy in reducing tumor growth. We advocate testing combination of targeted agents in a therapeutic strategy that move to prevention to address unmet clinical needs.

## Competing interests

The authors declare no conflict of interest.

## Authors’ contributions

CS: project conception, planned design and coordination of the project, inoculated the mice with tumor cells, performed radiological imaging and helped draft the manuscript. DB: project conception, planned design and coordination of the project, performed radiological imaging and helped draft the manuscript. SB: performed immunohistochemistry for TRAP and CD34, analysed the data. A-C.L: performed analyses of plain radiography images and tabulated data. DL: performed immunohistochemistry for TRAP, analysed the data. SH: performed sectioning of bone, immunohistochemistry for CD34, histomorphometry, data analyses and tabulation. FR: performed optical imaging, assisted in animal handling and dissection. HK: participated in conception, design and coordination of the project and helped draft the manuscript. CCG: participated in conception, design and coordination of the project and helped draft the manuscript. WJ: participated in conception, design and coordination of the project and helped draft the manuscript. ST: generated stable transfected cells, performed optical imaging, participated in data tabulation, analyses, interpretation of data and drafted the manuscript. All authors read and approved the final manuscript.

## Pre-publication history

The pre-publication history for this paper can be accessed here:

http://www.biomedcentral.com/1471-2407/13/32/prepub
